# Mechanism of isorhynchophylline in lipopolysaccharide-induced acute lung injury based on proteomic technology

**DOI:** 10.3389/fphar.2024.1397498

**Published:** 2024-05-30

**Authors:** Yaru Li, Junfeng Xing, Ling Qin, Chuanming Zhang, Zheng Yang, Min Qiu

**Affiliations:** ^1^ Department of Pharmacy, Baotou Medical College, Baotou, Inner Mongolia, China; ^2^ Department of Computer Science and Technology, Baotou Medical College, Baotou, Inner Mongolia, China; ^3^ First Affiliated Hospital of Baotou Medical College, Baotou, China

**Keywords:** TUNEL, RT-PCR, Nano-LC-MS/MS, isorhynchophylline, acute lung injury, proteomic technology

## Abstract

Isorhynchophylline (IRN), a tetracyclic indole alkaloid, has anti-inflammatory and antioxidant activities against cardiovascular diseases and central nervous system disorders. Acute lung injury (ALI) is a manifestation of inflammation concentrated in the lungs and has a high incidence rate and mortality The purpose of this study is to explain the mechanism of IRN in the treatment of acute lung injury and to provide a new scheme for clinical treatment. The experimental mice were divided into three groups: CTRL, LPS, LPS+IRN. The mouse model of ALI was established by inhaling LPS solution through nose. After continuous administration of IRN solution for 7 days, the mice in LPS+IRN group were killed and the lung tissue was collected for detection. Proteomic (Data are available via ProteomeXchange with identifier PXD050432) results showed that 5727 proteins were detected in mouse lung tissues, and 16 proteins were screened out. IRN could reverse the trend of these differential proteins. In addition, IRN can act on integrin αM to reduce neutrophil recruitment and thereby produce anti-inflammatory effects and may suppress neutrophil migration through the leukocyte transendothelial migration pathway. TUNEL and RT-PCR experiments revealed that LPS-induced ALI in mice increases the apoptosis of lung tissues, damage to alveolar epithelial cells and levels of inflammatory factors. Treatment with IRN can repair tissues, improve lung tissue pathology and reduce lung inflammation.

## 1 Introduction

Acute lung injury (ALI) refers to the primary diseases caused by various serious noncardiogenic pulmonary and extrapulmonary pathogenic factors, such as severe infection (sepsis), shock, trauma (mechanical ventilation-induced lung injury), disseminated intravascular coagulation, aspiration and fat embolism (Qian, 2003). The clinical manifestations are mainly acute progressive exacerbation of dyspnoea and refractory hypoxemia. The incidence and mortality rates of ALI are high, so finding drugs that can treat this condition is important. But at present, the drug treatment of ALI is mainly symptomatic treatment, relieving the symptoms caused by ALI through diuresis, anti-inflammation and antioxidation, anti-platelet aggregation, neuromuscular blockers and so on (Chen and Liu, 2021). Stem cell MSC in the treatment of lung injury is a new research hotspot, which can not only treat symptomatic, but also produce anti-inflammatory factors and a variety of paracrine factors on the basis of inhibition of pro-inflammatory factors, and can be induced to differentiate into alveolar epithelial cells *in vivo*. Bronchial epithelial cells promote tissue healing. However, up to now, MSC can only be extracted from bone marrow and umbilical cord, and its early preparation is complex and expensive, so its application is limited (Johnson and Matthay, 2010; Lee et al., 2011). Isorhynchophylline (IRN), a tetracyclic indole alkaloid, is one of the main active ingredients in plants of the Rubiaceae family. Previous research on IRN has mainly focused on its anti-inflammatory and antioxidant effects on the cardiovascular, cerebrovascular and central nervous systems (Deng et al., 2021; Zhou et al., 2013). After it was found that it had anti-inflammatory and antioxidant effects, the research on IRN was gradually extended to acute renal injury, osteoarthritis and lung injury (Zheng et al., 2021; Li et al., 2022). It was found that IRN can reduce the release of inflammatory mediators in lung tissue and alleviate the disease process of acute lung injury. However, the mechanism of IRN in treating ALI remains unclear. Therefore, this study uses proteomic techniques for analysing the changes in lung tissue proteins in mice with ALI to elucidate the pharmacological mechanism of IRN in treating ALI and provide a scientific basis for the use of IRN to ameliorate this condition.

## 2 Materials and methods

### 2.1 Chemicals and reagents

IRN was purchased from Macklin, and lipopolysaccharide (LPS) (O55: B5) was obtained from Biyuntian Biotechnology Co., Ltd. TUNEL reagent kit was acquired from Servicebio; SweScript All in One RT SuperMix for qPCR (One Step gDNA Remover) was bought from Wuhan Saiwei Biotechnology Co., Ltd. and 2× Universal Blue SYBR Green qPCR Master Mix was purchased from Wuhan Saiwei Biotechnology Co., Ltd. tandem mass tag (TMT) labelling reagents were provided by Thermo and COmplete™Mini protease inhibitor mixture and PhosSTOP™ phosphatase inhibitor mixture tablets were acquired from Roche.

### 2.2 Experimental animals

Thirty SPF-grade healthy male ICR mice (6–8 weeks old, body weight of 40 + 2 g) were purchased from Peking University [production licence number: SCXK (Beijing) 2022-0009]. The mice were raised in plastic cages, with a standard environment of 25°C ± 1°C and a 12/12-h light–dark cycle, allowing for free eating and drinking. After 1 week of adaptive feeding, the experiment was started. All experiments in this study were approved by the Experimental Animal Ethics Committee of Baotou Medical College (Baotou City Experimental Animal Ethics Committee) (certificate number 182021).

### 2.3 Experimental grouping and medication

**FIGURE 1:**
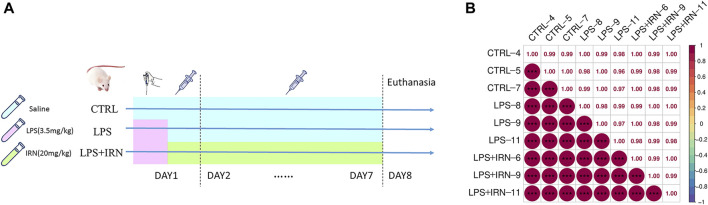
**(A)** Experimental plan. **(B)** Pearson correlation coefficient chart: The horizontal and vertical axes represent each sample, and the colour depth (bottom left part) or numerical size (top right part) represents the size of the correlation coefficient between the two samples. The closer to the red portion (the closer the correlation coefficient is to 1), the greater the correlation. The closer to the middle colour yellow (the closer the correlation coefficient is to 0), the smaller the correlation.

After 1 week of adaptive feeding, the 30 mice were randomly divided into three groups with 10 mice each: CTRL group, LPS model group and LPS + IRN treatment group. On the first day, all experimental mice were intraperitoneally injected with a tribromoethanol anaesthetic agent. After achieving complete anaesthesia, the mice were positioned on the table with their heads up and spines upright. In the LPS and IRN + LPS groups, the LPS solution (3.5 mg/kg in 50 μL) was absorbed with a pipette and placed near the nostrils of the mice. The mice inhaled the droplets remaining outside of the gun head; these steps were repeated for the other nostrils. For the mice in the CTRL group, the same procedure was followed, but with an equivalent volume of normal saline provided for inhalation. After these steps were completed, the mice were gently shaken to promote uniform distribution of the inhaled liquid in the trachea and lungs. After 4 h of administration, the CTRL and LPS model groups were intraperitoneally injected with 200 μL of physiological saline, and the LPS + IRN group was given the same volume of Isorhy solution prepared with physiological saline (20 mg/kg) for 7 consecutive days. At 24 h after the last administration, the mice were anesthetized and subjected to cervical dislocation to euthanise, and their lungs were taken. The specific experimental plan is shown in Figure 1A.

### 2.4 Isotope labelling relative and absolute quantitative TMT proteomics

The lower lobe of the right lung was stored at −80°C, and three samples were extracted from each group for analysis. The samples underwent protein extraction, quality control detection, TMT labeling after enzyme removal of salt, the peptide was separated by UltiMate™ 3,000 Binary Rapid Separation System. The separated peptide segments were detected by mass spectrometry, and protein libraries were finally searched.

### 2.5 TdT-mediated dUTP nick end labelling (TUNEL)

The upper lobe of the right lung was placed in 4% paraformaldehyde, stored at room temperature, fixed for more than 24 h, dehydrated, embedded in paraffin and cut into 4 μm-thick paraffin sections. The prepared paraffin sections were stained with TUNEL reagent kit, and the nuclei were restained with DAPI. Images were captured under a fluorescence microscope to analyse the apoptosis of cells in each group of samples.

### 2.6 Quantitative Reverse Transcription PCR (RT-qPCR)

The right middle lobe and posterior lobe of the lungs were stored at −80°C for RT-PCR detection of IL-1 β and Arg1 mRNA expression. RNA extraction lysis solution was applied to extract total RNA, strictly following the steps in the instructions of the reagent kit. A multifunctional UV-visible spectrophotometer was employed to determine RNA purity and concentration. mRNA reverse-transcriptase polymerase chain reaction was performed in the reaction system consisting of the following: 4 μL of 5 × SweScriptAll in one SuperMix, 1 μg of DNA remover, 10 µL of total RNA and 5 μL of deionized water. The reaction conditions were as follows: 25°C, 5 min; 42°C, 30 min; and 85°C, 5 s. Real-time fluorescence quantitative PCR amplification was conducted using cDNA as a template, and the reaction system comprised the following: 7.5 μL of 2XUniversalBlueSYBRGreenqPCR MasterMix, 4 μL of deionized water, 2.5 μM gene primers (upstream + downstream) and 2 μL of template cDNA. The amplification programme was as follows: 95°C, 30 s; 95°C, 15 s; 60°C, 30 s for 40 cycle. The fluorescence signal was collected once at 65°C–95°C at a rate of 0.5°C per litre. The melting curve was analysed, and the Ct value was interpreted based on the amplification curve. GAPDH was used as the internal reference, and the 2^−△△Ct^ method was adopted to calculate the relative expression level of the target gene. The primers were synthesised by Wuhan Seville Biotechnology Co., Ltd., and their sequences are shown in Table 1.

**TABLE 1:** Primer sequences for PCR.

Gene name	Primer information^a^	Primer sequence (5'-3')	Primer sequence (5'-3')
GAPDH	NM_008084.2	F: CCTCGTCCCGTAGACAAAATG	133
		R: TGAGGTCAATGAAGGGGTCGT	
IL-1β	NM_008361.4	F: GCTTCAGGCAGGCAGTATCA	185
		R: AATGGGAACGTCACACACCA	
Arg-1	NM_007482.3	F: CTGGGGATTGGCAAGGTGAT	93
		R: CAGCCCGTCGACATCAAAG	

^a^ Primers were designed from the published sequences in the GenBank database under the indicated accession numbers; F, forward primer; R, reverse primer.

### 2.7 Statistical analysis of data

The mass spectrometry proteomics data have been deposited to the ProteomeXchange Consortium via the PRIDE (Perez-Riverol et al., 2022) partner repository with the dataset identifier PXD050432.

The TUNEL results were obtained using ImageJ (National Institutes of Health), Bethesda (MD) grayscale analysis software was used for analysis, and experimental data results were expressed as mean ± standard deviation (mean ± SEM). The statistical software IBMSPS Statistics27 was used for one-way ANOVA and multiple comparison LSD *t*-test. All experimental data results were plotted using GraphPad 9.5 plotting software. The experimental images were edited using Adobe Photoshop 2020 *p* < 0.05 was considered statistically significant.

## 3 Experimental results

### 3.1 Effect of IRN on protein expression in lung tissue of mice with acute lung injury induced by LPS

#### 3.1.1 Sample correlation analysis

The clustering of samples can be determined by correlation analysis of the protein expression levels of samples. The larger the correlation coefficient between the samples, the better the clustering of samples and the higher the confidence of the proteomic data (Figure 1B).

#### 3.1.2 Differential protein screening results

**FIGURE 2:**
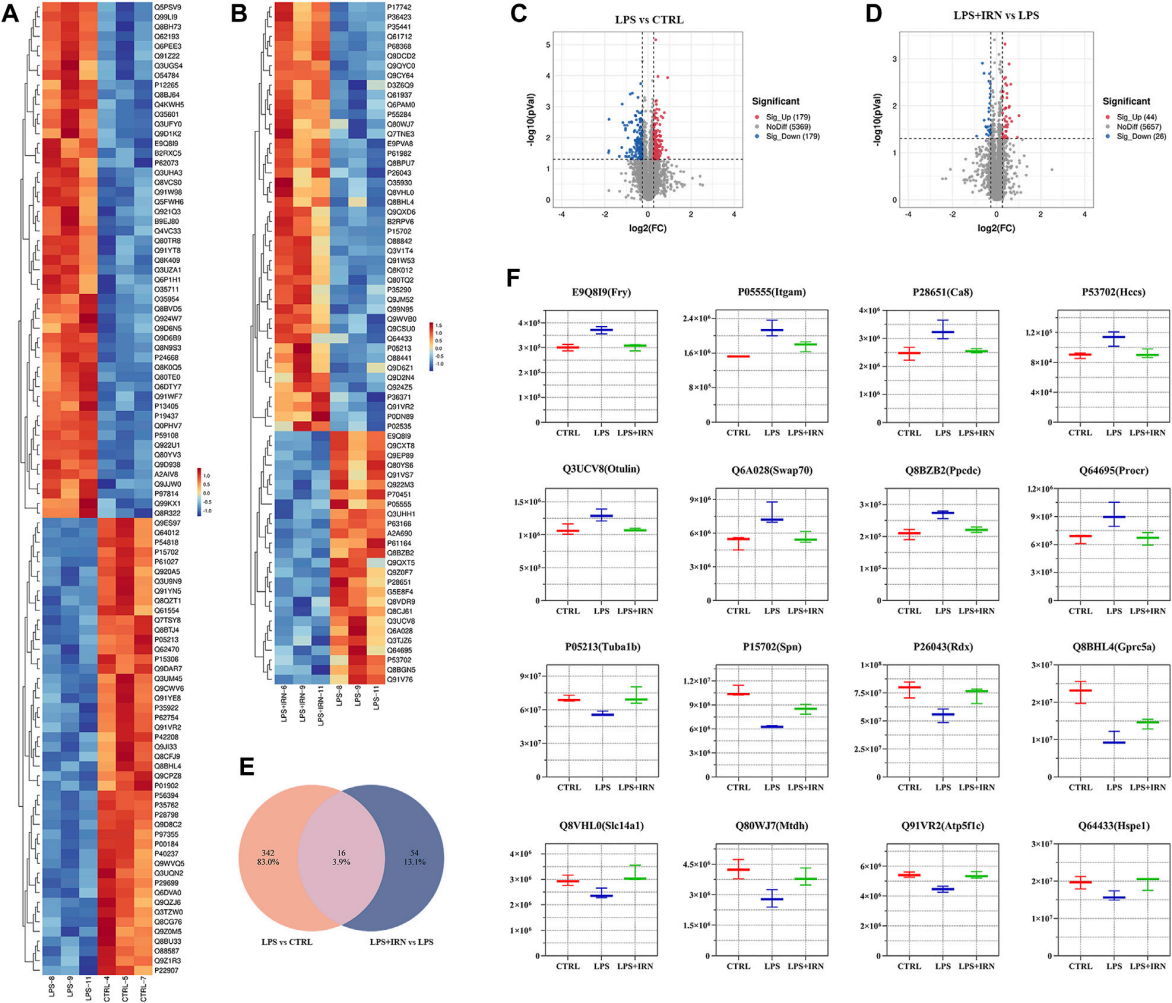
**(A,B)** Heat map: The horizontal axis represents the sample and the vertical axis represents the screened differentially expressed proteins (default is to take the Top100 protein with the lowest *p*-value or all differentially expressed proteins for heat map display). **(C,D)** Volcano plot: Using log2 (FC) as the *x*-axis and −log10 (*p*-value) as the *y*-axis, plot a volcano plot for all proteins in differential expression analysis. Among them, red represents significantly upregulated differentially expressed proteins, blue represents significantly downregulated differentially expressed proteins and grey dots represent nonsignificant differentially expressed proteins. **(E)** Wayne plot: Counting the intersection of differentially expressed proteins in each comparison group. **(F)** Box plot: The horizontal axis represents the group name, the vertical axis represents the protein abundance, and the graphic title is protein ID (corresponding gene name).

With the use of TMT proteomic quantitative technology, proteins were screened simultaneously from fold change (FC) and significance level (*p*-value). With difference multiple FC ≥ 1.2 or FC ≤ 0.83 (i.e., the absolute value of log2FC ≥ 0.263) and a *p*-value <0.05 (| log2FC | ≥ 0.263 & *p* < 0.05) as the threshold criterion, the screened genes were considered differentially expressed proteins. Compared with the CTRL group, 358 proteins were differentially expressed in the lung tissues of the LPS group, of which 179 were upregulated, and 179 were downregulated (Figures 2A, C). Compared with the LPS group, 70 proteins were differentially expressed in the lung tissues of the IRN group, of which 44 were upregulated, and 26 were downregulated (Figures 2B, D).

Through comparative analysis, it is found that 16 different proteins were same in the two comparison groups (Figure 2E). There were 16 different proteins that could reverse the difference trend after IRN intervention: compared with CTRL group, LPS group upregulated the expression of E9Q8I9 (Fry), P05555 (Itcam), P28651 (Ca8), P53702 (Hccs), Q3UCV8 (Otulin), Q6A028 (SWAP-70), Q8BZB2 (Ppcdc), Q64695 (Procr), and downregulated P05213 (Tuba1b), P15702 (Spn), P26043 (Rdx), Q8BHL4 (Gprc5a), Q8VHL0 (Slc14a1), Q80WJ7 (Mtdh), Q91VR2 (Atp5f1c), Q64433 (Hspe1). Compared with LPS group, LPS + IRN group reversed the change of protein expression induced by LPS (Figure 2F).

#### 3.1.3 GO enrichment analysis of differential proteins

To map the differential proteins between the two groups to the entries annotated in the protein Gene Ontology database, calculate the number of proteins in each item, and classify them, we made a Proteomic differential protein GO enrichment analysis.

**FIGURE 3:**
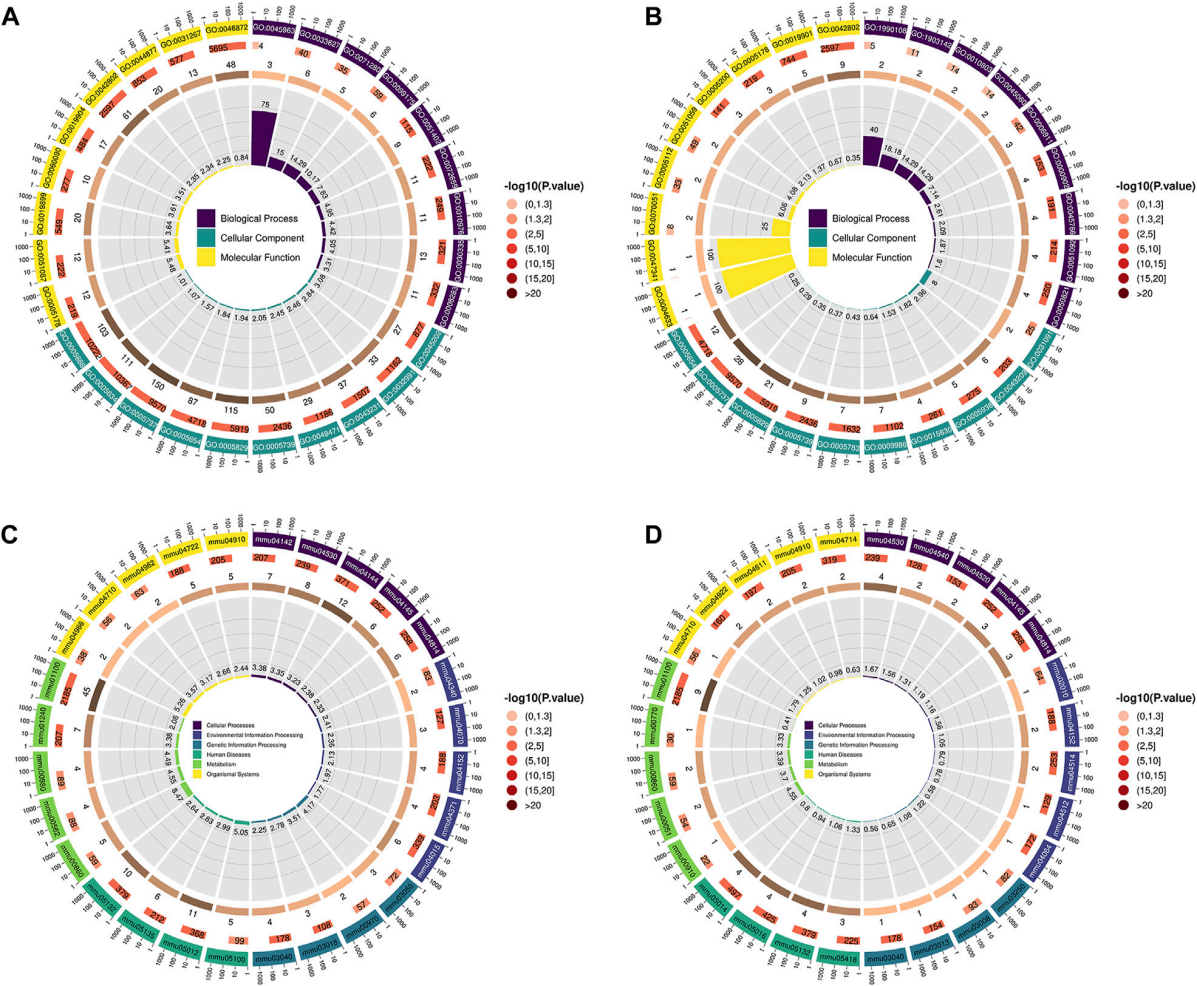
**(A,B)** GO enrichment analysis; the first circle (from outside to inside) shows the enriched top (with the lowest *p*-value) GO entry, the outer circle shows the coordinate ruler of protein number, and different colours represent the classification of the three major categories of GO. The second circle represents the number of proteins annotated to the GO entry, and the colour represents the −log10 value of the enrichment analysis P or Q value. The third circle shows the statistics of the number of differentially expressed proteins in GO, where the number represents the number. The fourth circle represents the percentage of enrichment factors (Rich. Factor). **(A)** LPS group VS CTRL group, **(B)** LPS + IRN group VS LPS group. **(C,D)** The first circle (from outside to inside) is the KEGG pathway enriched in the Top (with the lowest *p*-value), and the outer circle is the coordinate ruler of the number of proteins. Different colours represent different KEGG first-level classifications (KEGG Level 1). The second circle represents the number of proteins annotated to the KEGG pathway, and the colour represents the −log10 value of the enrichment analysis P or Q value. The third circle shows the statistics of the number of differentially expressed proteins in the KEGG pathway, where the number represents the number. The fourth circle represents the percentage of enrichment factors (Rich. Factor), where **(C)** represents the LPS group VS CTRL group and **(D)** represents the LPS + IRN group VS LPS group.

Compared with the CTRL group, the differential proteins in the lung tissues of the LPS group mice were significantly enriched in the following terms (Figure 3A). Integrin mediated cell adhesion for biological process (BP). Protein-containing complexes, intracellular binding organelles, perinuclear regions and mitochondria for cellular component (CC). And integrin binding, for molecular function (MF).

Compared with those in the LPS group, the differential proteins in the lung tissues of the LPS + IRN group were significantly enriched in the following terms (Figure 3B). Positive regulation of endothelial barrier establishment, signal transduction regulation mediated by tumour necrosis factor and selection of negative thymic T cells for BP. Platelets, α granules, cell cortex and microtubule cytoskeleton for CC. And fibrinogen binding for MF.

#### 3.1.4 KEGG enrichment analysis of differential proteins

The differential proteins between the two groups were divided into groups according to sequence similarity, and the proteins with similar functions on the same pathway were then labeled with KO (or K), and the proteomic differential protein KEGG enrichment analysis was performed.

Compared with those in the CTRL group, the differential proteins in the lung tissues of the LPS group were mainly enriched in the following signal pathways (Figure 3C). Bacterial invasion of epithelial cells, tight junctions, lysosomes, biosynthesis of cofactors, cytochrome P450 metabolism of exogenous drugs, metabolic pathways and endocytosis.

Compared with those in the LPS group, the differential proteins in the lung tissues of the LPS + IRN group were significantly enriched in the following signal pathways (Figure 3D): tight junction, fructose and mannose metabolism, porphyrin metabolism, *Salmonella* infection, and gap junction.

#### 3.1.5 Interaction network analysis of differential proteins

**FIGURE 4:**
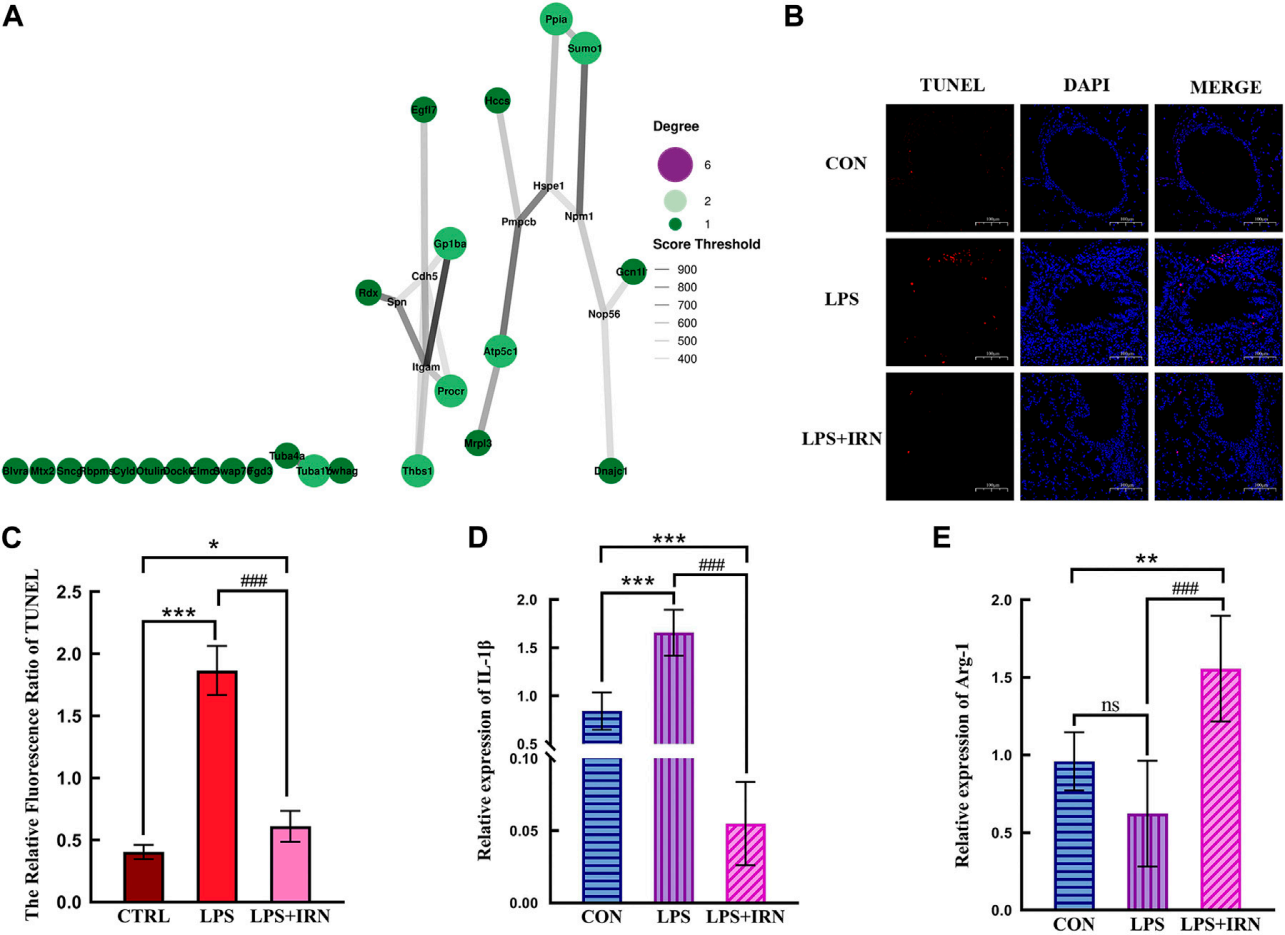
**(A)** The size of bubbles is related to connectivity. The higher the connectivity, the larger the bubbles and the higher their importance in the network. The line represents the correlation, and the thickness of the line indicates the strength of the correlation. The thicker the line, the stronger the correlation. **(B)** TUNEL status of lung tissue in each group (×100). **(C)** The Relative Fluorescsncs Ratio of TUNEL. **(D)** The relative expression level of IL-1β in lung tissue of mice in each group. **(E)** The relative expression level of Arg-1 in lung tissue of mice in each group. **(C,D,E)** * Compared with CTRL, # Compared with LPS; *, #*p* < 0.05, **, ##*p* < 0.01, ***, ###*p* < 0.001.

With the use of the STRING database combined with Cytoscape software, a PPI network diagram was constructed to compare the differential proteins between the LPS and IRN groups. Protein interaction pairs with a score greater than 400 were screened. As shown in Figure 4A, a correlation was found between the therapeutic effect of IRN on LPS-induced ALI and the functionality of Cdh5 and Itcam (mitochondrial processing peptide subunit beta, P55284 (Cdh5), integrin αM).

### 3.2 Effects of IRN on lung tissue apoptosis in LPS-induced acute lung injury model mice

A representative fluorescence microscope image is shown in Figure 4B. TUNEL fluorescence staining showed that compared with the CTRL group, the LPS model group had a significant increase in the red fluorescence area of TUNEL-stained lung tissue slices (*p* < 0.001), indicating an increase in apoptotic cells. Compared with that in the LPS model group, the red fluorescence area (apoptotic cells) stained with TUNEL in the lung tissue slices of the LPS + IRN group was significantly reduced (*p* < 0.001), indicating that IRN can reduce the apoptosis of lung tissue and lung cells in mice with LPS-induced ALI (Figure 4C).

### 3.3 Effects of IRN on the expression of IL-1β and Arg-1 in lung tissues of mice with LPS-induced acute lung injury

Whole RNA was extracted from lung tissues for RT-PCR detection. LPS significantly increased IL-1β mRNA expression level in mouse lung tissues compared with that in the CTRL group (*p* < 0.001) (Figure 4D). Meanwhile, IRN can significantly inhibit IL-1β mRNA expression (*p* < 0.001). The mRNA expression level of IL-1β was significantly lower than that of the CTRL group mice (*p* < 0.001). Compared with that of the CTRL group, no significant difference in the mRNA expression level of Arg1 was observed for the LPS model group (*p* > 0.05). Meanwhile, the mRNA expression level of Arg1 in the LPS + IRN group significantly increased (*p* < 0.01) (Figure 4E).

## 4 Discussion

The pathogenesis of ALI is complex and intricate, with its physiological basis being the disruption of the vascular endothelial alveolar epithelial barrier, which is essentially an uncontrolled inflammatory response (Tao et al., 2016). The main pathogenesis includes oxidative imbalance, inflammatory response imbalance (Th1 and Th2 ratio imbalance), cell apoptosis, imbalance of coagulation and fibrinolysis system and pulmonary oedema.

IRN is one of the main active ingredients and is widely used in traditional Chinese medicine. It has anti-inflammatory effects on glial cells, acute kidney injury and other conditions (YUAN et al., 2009; Chen et al., 2010; Zheng et al., 2021; Li et al., 2022). Research on the treatment of lung diseases with IRN has gradually increased. Past studies have reported that IRN has a therapeutic effect on asthma because it reduces the release of airway inflammatory mediators through different signalling pathways, such as MCP-1/CCR2 and MIR-200a-mediated Foxc1/NF-κB. This leads to anti-inflammatory and antioxidant effects that alleviate lung injury and asthma symptoms (Zhu et al., 2020; Hou et al., 2021; Cai et al., 2023). Chen et al. (2022) reported that IRN could alleviate bleomycin-induced pulmonary fibrosis in mice, mainly by regulating the ERK/p27 signalling pathway. Qiu et al. (2020) reported that IRN possesses a protective effect against lung injury induced by silica. Additionally, IRN pretreatment was found to inhibit oxidative stress responses in mouse alveolar macrophages induced by lipopolysaccharide, reducing the anti-inflammatory and antioxidant effects in endotoxin-stimulated mouse alveolar macrophages (Zhou et al., 2019).

This study used proteomic detection methods to observe the effect of IRN on LPS-induced ALI in mice to predict its mechanism as a treatment for this condition. GO enrichment analysis and KEGG enrichment analysis of differential proteins between the LPS and CTRL groups showed that LPS inhalation induced endothelial cell barrier disruption, which is closely related to the increased neutrophil adhesion and cell apoptosis in the pathogenesis of ALI and is consistent with previous research on the mechanism of ALI (Johnson and Matthay, 2010). GO enrichment analysis and KEGG enrichment analysis of differential proteins between IRN and LPS groups showed that IRN can regulate microtubule cytoskeleton, increase cell proliferation, positively regulate endothelial barrier, repair cell and tissue functions and increase cell fibrinogen regulation, thereby regulating bleeding/coagulation balance and tumour necrosis factor-mediated model transmission and reducing inflammation. KEGG signalling pathway analysis also showed that the differential proteins of IRN were enriched in cardiovascular diseases and neurodegenerative diseases, which is consistent with previous research on IRN in cardiovascular diseases and glial inflammation (Deng et al., 2021; YUAN et al., 2009), further proving that IRN has a similar pathway in treating ALI and confirming its anti-inflammatory and antioxidant functions in ALI.

Differential protein analysis revealed that IRN can reverse the expression of 16 differentially expressed proteins caused by LPS (Figure 2F). LPS upregulates P05555 (Itcam), Q64695 (Procr), Q8BZB2 (Ppcdc) and Q6A028 (SWAP-70), all of which can promote neutrophil aggregation and migration, thereby producing inflammatory factors and amplifying the inflammatory response. LPS upregulates Q3UCV8 (Otulin), activates T cells and promotes the occurrence and development of inflammatory responses (Gubser et al., 2013; Cammann et al., 2016; Kim and Suresh, 2013). It also downregulates P15702 (Spn), Q8BHL4 (Gprc5a), Q80WJ7 (Mtdh), P05213 (Tuba1b) and Q64433 (Hspe1) proteins, reducing the restriction on inflammatory factors and providing a favourable environment for the occurrence and development of inflammation (Cannon et al., 2011; Allenspach et al., 2001; Cannon et al., 2008; Damgaard et al., 2016; Heger et al., 2018; Damgaard et al., 2016; Mody et al., 2007). LPS downregulates P26043 (Rdx) and Q91VR2 (Atp5f1c), which can enhance the occurrence and development of inflammatory reactions in the body. The accumulation of these factors ultimately leads to an outbreak of inflammation. IRN can reverse the expression of these proteins, thereby reducing the adhesion and aggregation of neutrophils, limiting the transmission of inflammatory signals and the occurrence and development of inflammatory reactions and producing anti-inflammatory effects. It also enhances the expression of Q8VHL0 (Slc14a1), which can promote tissue repair (Rivkin et al., 2013).

In protein–protein interactions (Figure 4A), P55284 (Cdh5) and P05555 (Itgam) are key proteins for IRN to function. P55284 (Cdh5) is a calcium-dependent cell adhesion protein. This type of cadherin plays an important role in endothelial cell biology by controlling the cohesion of intercellular connections and tissue (Lampugnani et al., 2010; Matsuyoshi et al., 1997). P05555 (Itgam) participates in various adhesion interactions among monocytes, macrophages and granulocytes, regulating neutrophil migration. It also enhances the adhesion among white blood cells, inflammatory cells and endothelial cells; promotes neutrophil recruitment and endothelial cell activation; and has the same influence as P55284 (Cdh5), resulting in reduced connections between endothelial cells and alveolar epithelial cells, leading to the destruction of endothelial cell integrity and making it easy to penetrate the endothelium (Zhan et al., 2011; Gong et al., 2014; Li and Ding, 2008; Zheng et al., 2016). LPS destroys the vascular endothelial barrier by down-regulating P55284 (CDH5), and upregulates P0555 (ITGAM) to enhance the recruitment and activation of inflammatory cells, resulting in the occurrence and development of inflammation. IRN enhances intercellular connections and repairs the alveolar epithelial cell barrier by regulating P55284 (Cdh5), and downregulate P0555 (ITGAM) to reduce neutrophil recruitment and play an anti-inflammatory effect.

This experimental study found that the alveolar structure of the CTRL group was intact, and the epithelial cells were arranged neatly (Figure 4B). When inhaled, LPS can cause the increased apoptosis of alveolar and bronchial epithelial cells, early destruction of alveolar integrity and thickening of alveolar spaces. These pathological phenomena are related to proteomic analysis, where LPS induction leads to a decrease in the expression of P55284 (Cdh5) cadherin. The expression of inflammation-related factor IL-1β significantly increased (*p* < 0.001) (Figure 4D), which was related to the increased expression levels of differential proteins P05555 (Itcam), Q64695 (Procr), Q8BZB2 (Ppcdc) and Q6A028 (SWAP-70) in the proteomic analysis. The decrease in the expression levels of proteins related to limiting inflammatory response, such as P15702 (Spn), Q8BHL4 (Gprc5a), Q80WJ7 (Mtdh), P05213 (Tuba1b) and Q64433 (Hsp1), further increased the intensity of inflammatory response. A comparison between the LPS + IRN and LPS groups revealed that apoptosis was reduced, and alveolar structural integrity and alveolar space were restored after IRN administration (Figure 4B). The expression of the inflammation-related factor IL-1 β was significantly decreased (*p* < 0.001), while the expression of Arg1 was significantly increased (*p* < 0.01) (Figures 4D, E). Proteomic analysis indicated that IRN could reverse the expression of these proteins and increase the expression of Q8VHL0 (Slc14a1). IL-1β, a main pro-inflammatory factor, is a marker of inflammatory initiation in M1 macrophages. Arg1, an anti-inflammatory factor, is also a marker of M2 macrophages. The balance of macrophage types plays an important role in inflammation. In RT-PCR analysis (Figures 4D, E), the IL-1β expression in the LPS group was significantly higher than that in the CTRL group (*p* < 0.001), indicating that LPS activated inflammatory responses and initiated an inflammatory cascade reaction in mouse lung tissues. No significant difference was observed in Arg1 expression between the CTRL and M1 groups, implying that macrophages may be polarised to the M1 type. After the administration of IRN, compared to the LPS group, the LPS + IRN group showed significantly decreased pro-inflammatory factor IL-1β expression (*p* < 0.001) and decreased inflammatory reaction, while the expression of anti-inflammatory factor Arg1 increased (*p* < 0.001), suggesting may increased M2 macrophage polarization. On the basis of the above experimental results, IRN can inhibit the release of inflammatory factors and inflammation, increase the release of anti-inflammatory factors, and promote damage repair and tissue regeneration. In addition, IRN can induce macrophages to transition from M1 polarisation to M2 polarisation.

This study performed proteomic analysis to detect 16 differentially expressed proteins, which are mainly related to the integrity of the endothelial barrier and alveolar epithelial barrier, neutrophil recruitment, restriction of inflammatory response and damage repair. Based on previous research on IRN in cardiovascular and neurological diseases, we speculate that IRN may also have anti-inflammatory and antioxidant effects on ALI. Proteomic analysis suggested that the anti-inflammatory and antioxidant activities of IRN may be related to its regulation of neutrophils. On the one hand, IRN acts on neutrophils themselves. IRN can act on P05555 (Itgam), which hinders neutrophil adhesion to endothelial cells, reduces neutrophil recruitment and thus minimises the generation of inflammatory factors. On the other hand, IRN acts on the environment in which neutrophils exert their biological functions. IRN acts on protein P55284 (Cdh5), which is involved in leukocyte cross endothelial migration (leukocyte). The transmission pathway reduces cell apoptosis, repairs the endothelial barrier, and suppresses neutrophil migration to the lung interstitium and alveoli, thereby narrowing the diffusion range of inflammatory factors. In addition, IRN can enhance the immune system’s response to inflammation, thereby strengthening the restriction of inflammatory response and reducing damage to the body. Further verification using TUNEL and RT-PCR revealed that IRN can repair the integrity of alveolar epithelial cells, may induce macrophage polarisation towards M2 type, alleviate inflammatory response, produce anti-inflammatory activity and repair damage effects and further strengthen the evidence. At the same time, it is not clear which part of the above mechanism plays a leading role in IRN treatment of LPS-induced acute lung injury in mice in this study, and we will continue to investigate it in the following work.

## 5 Conclusion

Proteomics can be used to analyse the mechanism of IRN in treating ALI in mice. IRN can reverse the abnormal expression of related proteins in pathological processes, induce macrophage polarisation towards the M2 type, alleviate inflammation and repair damaged tissues. This study presents a new research direction for the treatment of LPS-induced ALI with IRN and provides a scientific basis for its clinical application.

## Data Availability

The datasets presented in this study can be found in online repositories. The names of the repository/repositories and accession number(s) can be found in the article/Supplementary material.
